# Dietary Patterns, Physical Activity, and Lifestyle in the Onset, Prevention, and Management of Noncommunicable Diseases

**DOI:** 10.3390/nu15112540

**Published:** 2023-05-30

**Authors:** Ronan Lordan, William B. Grant

**Affiliations:** 1 Institute for Translational Medicine and Therapeutics (ITMAT), Perelman School of Medicine, University of Pennsylvania, Philadelphia, PA 19104-5158, USA; 2 Sunlight, Nutrition, and Health Research Center, P.O. Box 641603, San Francisco, CA 94164-1603, USA; williamgrant08@comcast.net

Noncommunicable diseases (NCDs) are on the rise due to population growth and aging, which will cause a significant burden on global health systems [1]. Noncommunicable diseases account for most deaths worldwide. However, decades of research show that dietary and lifestyle factors play a significant role in the onset and progression of many noncommunicable diseases, including cardiovascular diseases (CVD) and cancers [2,3,4,5]. Furthermore, NCDs may be risk factors for communicable disease severity as is the case for the Coronavirus disease 2019 (COVID-19) [6,7], or indeed NCDs can increase the risk of developing other NCDs leading to individuals with many comorbidities. Therefore, there is an increased awareness in the development of programs addressing maladaptive dietary practices and lifestyles and increased research into how these factors may affect the onset, prevention, and management of NCDs. On the other hand, regular exercise is known to be beneficial for the prevention of NCDs and as an intervention for those who develop some NCDs such as CVD, type II diabetes mellitus, or obesity.

The aim of this Special Issue was to collate state-of-the-art original research articles and reviews addressing the role of dietary patterns, physical activity, and lifestyle in the onset, prevention, and management of NCDs. Several articles in the Special Issue investigated the role of dietary patterns in association with NCDs.

Hlaing-Hlaing et al. [8] investigated the association between the incidence of NCDs with diet quality measured using the alternative healthy eating index 2010, in an Australian longitudinal study of women’s health. Their repeated cross-sectional multivariate logistic regression analysis over a 15-year period showed that baseline diet quality does not demonstrate early evidence of a protective effect against a future occurrence of NCDs or multimorbidity between the aged of 25–45 years. Further longitudinal and temporal analyses with sensitive markers for NCD risk factors are required.

Wang et al. [9] conducted a prospective cohort study that investigated the association between fruit intake and stroke with consideration for genetic disposition in a cohort of almost 35,000 individuals from the project Prediction for Atherosclerotic Cardiovascular Disease Risk in China (CHINA-PAR). This study reported that a greater amount of fruit consumption was associated with a lower risk of stroke (28–32%), whereby individuals who had a higher genetic risk of stroke may gain more benefits in terms of more stroke-free years and absolute risk reduction. These findings add to the body of evidence demonstrating the importance of following a healthy dietary pattern for the prevention of stroke [10].

A cross-sectional study of over 40,000 participants was conducted by Zhu, et al. [11], who analyzed the association between obesity and dyslipidaemia using data from the Shanghai suburban adult cohort and biobank study (SSACB). Their data shows that central obesity and to some extent general obesity were associated with lower levels of high-density lipoprotein cholesterol (HDL-C) and higher levels of triglycerides. Other factors such as gender, age, diabetes, and hypertension were also associated with various forms of dislipidaemia. Indeed, these findings indicate the importance of further research into effective interventions for obesity to reduce the risk of metabolic syndrome and potential cardiovascular diseases due to the high prevalence of dyslipidaemia.

On the other hand, young adults are a population less explored in nutrition research. Kurniawan et al. [12] conducted a study with 1-year follow-up to determine the effects of urbanization on urban or rural individuals and their metabolic profiles. Baseline urban (*n* = 106) and rural (*n* = 83) participants were young (16–25 years old) female Indonesian adults, some of which were examined in a 1-year follow-up (*n* = 81 and *n* = 66 participants, respectively). The urbanization of individuals who previously resided in rural areas appeared to be associated with less favorable changes in adipokine profiles and adiposity. These findings indicate that migrating from rural areas to urban areas may lead to maladaptive changes in lifestyle that affect health.

Understanding the mechanisms of how various lifestyle factors affect the onset and development of NCDs is important for developing prophylactics and therapeutics. Harishkumar et al. [13] reviewed the specific nutrients that may impart antithrombotic and antiatherosclerotic biological activity against proinflammatory signaling induced by platelet-activating factor (PAF) via its receptor (PAF-R). They report the in vitro, ex vivo, and nutritional evidence regarding the potential benefits of consuming various nutrients including vitamins, trace metals, polar lipids, and other micronutrients, which are constituents of healthy dietary patterns such as the Mediterranean diet. They focus on the potential cardiovascular benefits that may be derived from targeting the PAF signaling cascade and discuss the need for more research in the field, which has been echoed by others in the field studying the role of dietary components in PAF-induced atherosclerotic diseases [14].

Liu et al. [15] examined representative data from the youth risk behavior surveillance system (YRBSS) study database of over 73,000 adolescents in the United States. They showed that the prevalence of suicidal ideation and planning was higher among adolescents that did not meet optimum 24 h movement guidelines set forth by the YRBSS study or soft drink consumption ≥ 3 times/day was associated with increased suicidal ideation, planning, and attempts with/without medication. However, cautious interpretation is required as a causal relationship cannot be inferred from a cross-sectional design and most responses were self-reported introducing recall biases. However, the findings should give pause to policy makers generally about the importance of a healthy lifestyle pattern to prevent suicidal ideation, planning, and attempts [16,17].

Vitamin D research is also featured as a prominent element of this Special Issue. Grant and Boucher [18] reviewed how the seasonal varations of atomospheric temperature, humiduty, and solar radiation exposure affect seasonal variations of blood pressure, CVD rates, and respiratory viral infections. This review points to the importance of safe sun exposure in health and disease and the need for further public health research on the topic.

Two reviews in this Special Issue focused on the role of vitamin D-related risk factors for both maternal morbidity [19] and mortality [20]. Vitamin D deficiency has been previously associated with adverse pregnancy outcomes [21]. The first article conducted a systematic review of studies relating to vitamin D status during pregnancy and maternal outcomes [19]. The second article examined both morbidity and mortality as part of meta-analyses of the same topic. The authors show that vitamin D supplementation may be important for reducing the risk of pregnancy complications such as gestational diabetes, hypertension, preeclampsia, and others, and that low-circulating 25-hydroxyvitamin D [25(OH)D] concentrations during pregnancy were a risk factor for these complications [20]. These articles highlight the importance of vitamin D in the health of the mother and fetus during pregnancy.

The final two articles of the Special Issue address findings relating to the Coronavirus disease 2019 (COVID-19) pandemic and vitamin D. Vitamin D has been postulated as a potential prophylactic and therapeutic for COVID-19. Gupta, et al. [22] carried out case–control studies within two institutions of the University of California San Diego (UCSD) health system. They measured 25(OH)D concentrations in patients either prior to or post disease diagnosis within 180 days of diagnosis in January 2020. The authors reported that serum 25(OH)D status was not associated with an increased risk of COVID-19, but concentrations were reduced post infection. These findings provide further evidence that serum 25(OH)D concentrations measured after COVID-19 should not be used to determine the risk of COVID-19 with respect to 25(OH)D concentration but might be useful in predicting prognosis afterwards.

Post acute sequalae of COVID-19 (PASC) or more commonly known as long COVID-19 has emerged as a burdening health risk of SARS-CoV-2 infection. Barrea, et al. [23] conducted a review of the literature to determine if vitamin D levels play a role in long COVID. They surmise that vitamin D may play a role in long COVID due to its immunomulatory effects and its association with COVID-19 severity. A recent review by Moukayed [24] supports the findings of Barrea et al. [23] and suggests that higher doses of vitamin D supplementation may improve the health and survivorship of COVID-19 and long COVID-19 patients. However, further research is required to determine the role of vitamin D in long COVID aetiology and pathology.

This Special Issue has captured the breadth of research being undertaken regarding dietary patterns, physical activity, and lifestyle in the onset, prevention, and management of noncommunicable diseases.
